# Supplementary material for: Are carbon emissions trading and green financial instruments synergistic? -Comprehensive quantitative research based on content analysis
Source: PLoS One. 2024 Mar 7;19(3):e0298601. doi: 10.1371/journal.pone.0298601 (PMC10919601; doi:10.1371/journal.pone.0298601)
Supplement: S1 Data — (ZIP) [file pone.0298601.s001.zip › Data/地级市Z_F工作报告.zip!/║╙─╧/╨φ▓2╩╨/└┤╫╘╣1⁄2╓┌║┼í╛┬φ┐╦╩2╛▌═°í┐_2017.docx]

各位代表：

现在，我代表市人民政府，向大会报告工作，请予审议，并请各位政协委员和其他列席人士提出意见。

一、过去五年工作回顾

过去的五年，是我市发展进程中极不平凡的五年。五年来，在省委、省政府和市委的正确领导下，全市上下深入贯彻习近平总书记系列重要讲话精神，认真践行五大发展理念，拉高标杆，做强产业，两手发力，加快发展，全面完成了市六届人大确定的目标任务，取得了令人鼓舞的重大成就，极大激发了全市人民的自信心和自豪感。

——综合实力大幅提升。2016年，全市生产总值完成2353.1亿元、居全省第4位，人均生产总值5.4万元，均是2011年的1.6倍；一般公共预算收入131.9亿元，是2011年的1.8倍；固定资产投资2263.8亿元，年均增长20.3%。许昌综合实力稳居全省第一方阵。

——创新活力持续迸发。2016年，专利申请量和授权量分居全省第2位、第3位，分别是2011年的4.1倍和3倍。省级工程技术研究中心总量居全省第3位，企业研发平台数量较2011年翻了一番；科技进步对经济增长的贡献率达到59%，连续6次荣获全国科技进步先进市称号。

——民营经济蓬勃发展。全市民营经济增加值约占经济总量80%，比2011年提高近5个百分点，有效激发了经济发展活力和竞争力。民营企业达到5.1万家、从业人员156万，占全市就业总量90%以上。主营业务收入超百亿元民营企业5家，其中4家连续3年入围“中国民营企业500强”。

——发展后劲不断增强。郑许融合上升为省级战略，许昌在全省大局中的地位和作用更加凸显。基础设施不断完善，石武客专建成通车，郑万、郑合高铁和禹亳铁路开工建设，南水北调中线工程许昌段顺利通水。新兴产业加速扩张，传统产业稳步升级，森源电动汽车产业园、中原电梯产业园、许继智能电网装备制造基地等，一大批带动结构调整和转型升级的重大项目相继实施。土地、资金、人才、技术、信息和平台等生产要素得到进一步优化配置，支撑我市经济平稳较快发展的有利条件和积极因素不断累积。

——城乡面貌焕然一新。“三大水利项目”竣工投用，“五湖四海畔三川、两环一水润莲城”的水系景观惊艳呈现，许昌由一个严重缺水城市嬗变为“北方水乡”。城市建成区绿化覆盖率达到40.62%，绿色已成为许昌的鲜亮底色。“美丽乡村”建设富有实效，神垕镇成为国家级特色小镇，4个镇被列为全省首批重点示范镇。成功摘取全国文明城市桂冠，成为唯一一个既在全国创建文明城市工作经验交流会上作经验介绍，又被中央媒体集中巡礼报道的地级市。成功创建国家节水型城市、国家水生态文明城市，顺利通过国家卫生城市、国家园林城市复检，在全省唯一获评2016年度全国质量魅力城市。大美许昌，正在从愿景走进现实。

——人民生活继续提高。城乡居民人均可支配收入分别达到27016元、14357元，年均分别增长9.6%和10.5%。累计新增城镇就业46.3万人，完成农村劳动力转移就业47.5万人。建设各类保障性住房6.26万套，城乡低保、农村五保对象实现应保尽保，172个贫困村、19.44万贫困人口稳定脱贫，人民群众更加幸福、更有尊严。

五年来，我们主要做了以下工作：

（一）把稳增长作为首要任务，持续精准发力，经济发展保持良好态势。面对复杂严峻的经济形势，制定实施了稳增长保态势一揽子政策措施，打好政策“组合拳”，全市经济保持稳定增长，主要经济指标增速均高于全省平均水平。突出稳工业，设立资金池调贷，协调金融机构增贷，引导企业上市融资，帮助企业拓宽融资渠道，为企业提供实实在在支持，全市规模以上工业增加值、主营业务收入、利润总量均居全省前3位，境内主板上市企业5家，新三板挂牌企业23家，区域股权市场挂牌企业43家。加快发展现代服务业，服务业增加值达到815.3亿元、年均增长11.5%，对经济增长的贡献率达到40.5%，服务业占比提高7.8个百分点。加快转变农业发展方式，粮食生产能力稳步提高，培育省级以上农业产业化龙头企业43家、省级农业产业化集群9个，特色农业和都市生态农业发展成效显著。积极扩大有效投资，累计谋划实施重大项目741个、完成投资3118亿元，带动全市固定资产投资8314.7亿元。2016年全市生产总值增长8.8%，固定资产投资增长17.2%，增速均居全省第1位。

（二）把调结构作为主攻方向，推进转型升级，新经济新动能加快形成。制定实施中国制造2025许昌行动纲要、战略性新兴产业培育发展计划、高新技术企业培育发展计划和“互联网+”行动计划，促进产业结构向中高端迈进，工业竞争力居全省第3位。提升发展制造业，装备制造业集群规模达到1742亿元，对规模以上工业的贡献率超过50%，其中智能制造占比超过40%。培育壮大战略性新兴产业，形成战略性新兴产业核心集聚区，产值占全省15%以上。发挥科技创新引领作用，建成国家级科技企业孵化器、科技大市场等创新平台，国家级高新技术企业由2011年的22家增至80家，规模以上高新技术产业增加值占规模以上工业增加值比重达40.6%，成为全省首批“双创”示范基地。以电子商务为代表的新业态迅猛发展，全市应用电子商务企业3900家、年交易额达到409.6亿元。加快现代物流业发展，成为全国二级物流园区布局城市，相继建成众品冷链物流园、中原农产品物流基地等大型物流园区。大力发展现代金融业，金融机构达到117家，入选国家产融合作试点城市，连续2次荣获全省优秀金融生态省辖市。加快载体建设和提质发展，全市10个省级产业集聚区、5个省级服务业“两区”全部达到星级标准。

（三）把城镇化作为重要引擎，统筹城乡发展，城镇化质量持续提升。强化规划引领，新一轮城市总体规划获批实施，城乡规划体系逐步完善。着力提升中心城区首位度，中心城区建成区面积达到108平方公里，人口96万。累计实施城建重点项目754个、完成投资656亿元。城区道路、广场、游园、公厕等基础设施不断改造完善，绿化、亮化、美化工作持续巩固提升。市城乡一体化示范区核心区建设初见雏形，发展成效开始显现。许昌县撤县设区，城乡发展空间格局得到优化。中心城区至组团县（市）城际公交全面开通，成为全省首批公交优先示范城市。“五城联创”全面启动，海绵城市建设顺利推进，城市管理3个项目荣获“中国人居环境范例奖”。中心城区和5个县（市）全部入围省百城建设提质工程，城市发展“1+5”PPP投融资协议成功签约，被确定为全国重大市政工程领域PPP创新工作重点城市。新农村建设取得实效，改造提升农村公路2100公里，行政村通车率100%，改造危房7240户，新增农村自来水受益人口126万人。全市城镇化率达到49.38%，比2011年提高8.46个百分点，城镇的承载能力、产业吸纳就业能力显著增强，在新型城镇化“赶考”中，交出了一份发展势头好、城乡变化大、群众得实惠的答卷。

（四）把抓改革作为根本之策，扩大对外开放，发展活力更加强劲。深入推进供给侧结构性改革，扎实开展“三去一降一补”专项行动，关闭退出煤矿19处、压减产能397万吨，商品房去化周期降至合理区间，企业减税政策全面落实。国企改革基本完成，事业单位分类改革、财税体制改革稳步推进，营改增改革顺利实施。商事制度改革加快推进，“五证合一”改革全面启动，在全省率先颁发不动产登记证书。大力推进“放管服”改革，公共资源交易平台全部整合，保留行政审批事项全省最少，权力清单和责任清单全面推行，市县两级政府机构改革、公务用车制度改革顺利完成。国家中医药综合改革试验区获批建设，市中心医院深化改革顺利完成，县级公立医院综合改革平稳推进。全国完善建设用地使用权二级市场试点获批，农村、环保、城管等改革扎实有效。实施招商引资行动计划，坚持每季度在沿海发达地区举办专题招商活动，三国文化旅游周、花博会和药交会等市内招商活动成效显著，累计引进省外资金项目1848个、实际到位省外资金1802亿元。积极对接“一带一路”战略，加快“走出去”步伐，与德国、东盟等国家和地区交流合作不断加深，中德（许昌）产业园上升为省级战略，再生金属生态城、年产80万吨精密不锈钢连轧等一批项目落地建设，累计落地境外资金项目57个、实际利用外资29.2亿美元。积极扩大对外贸易，出入境检验检疫局、海关等开放平台设立运行，进出口总值增速居全省前列。

（五）把惠民生作为根本目的，顺应群众期待，城乡居民生活质量进一步提高。坚持每年办成一批民生实事，全市财政民生支出累计806亿元，占财政支出的72.2%。扎实开展脱贫攻坚，实现低保线、贫困线“双线合一”。合力打好大气污染防治攻坚战，突出重点源头、重点行业、重点区域污染治理，去年全市优良天数达到226天，居全省第3位。清潩河五年水变清目标提前实现，省定出境断面水质综合达标率100%，全省最严格水资源管理制度考核位居第一。着力扩大就业创业，就业保持稳定增长，成为全省唯一荣获“全国创业先进城市”的省辖市。各级各类教育协调发展，累计新增幼儿园407所，新建改扩建中小学123所，学前教育三年毛入园率、九年义务教育巩固率、高中阶段毛入学率分别达到91.8%、100%、92%，规划建设职教园区，在全省率先实现职教强县全覆盖，全面启动基础教育提升三年攻坚行动计划。四级医疗和公共卫生服务网络不断健全，中医服务体系进一步完善，“十五分钟健康服务圈”基本形成。“全面两孩”政策平稳实施，2016年全市人口自然增长率稳定在6.21‰。城乡公共文化服务体系日益完善，文化市场健康发展，城市文化软实力显著提升。城乡低保月人均补助水平分别提高到不低于260元和137元，农村五保对象集中供养、分散供养标准分别提高到每人每年不低于4000元和3000元。城乡居民医保完成整合，大病保险制度全面实施，城乡居民社会养老保险参保率达到98.8%，全覆盖的养老保险体系、社会救助体系基本建立。市、县公共自行车系统互联互通，三级农产品市场体系建成投用。实施棚户区改造项目74个，建设面积385万平方米，11.2万人住房条件明显改善。保障性安居工程有序实施，为更多困难家庭圆了“安居梦”。人民群众幸福感、获得感不断增强。

（六）把促和谐作为重要职责，防范化解风险，社会大局保持稳定。深入推进依法治理、平安建设，科学预判、妥善处置各类风险，非法集资、问题楼盘、信访积案三类突出问题基本得到有效化解。切实防范金融风险，严厉打击逃废银行债务行为，化解企业财务风险资金490笔44.5亿元，金融业保持稳定有序发展。保持打非治违高压态势，深化重点行业领域治理整顿，连续5年未发生重特大事故，全市安全生产形势总体平稳。严厉打击食品药品违法活动，积极创建国家食品安全示范城市，465家餐饮单位实施“互联网+明厨亮灶”工程。强化社会治安综合治理，依法打击各类违法犯罪活动，连续3次荣获全省平安建设先进市，2016年全市经济发展、行政、法制3项环境指数均居全省第1位，人民群众安全感不断提升。

五年来，我们自觉接受人大、政协和社会监督，认真办理人大代表议案、建议和政协提案，积极支持民主党派、工商联和无党派人士参政议政。大力推进依法行政，加强政府立法和法治宣传教育，“六五”普法圆满完成，建立完善政府法律顾问、“三重一大”决策等制度。扩大政务公开，切实加强行政监察和审计监督，着力打造阳光政府。深入开展党的群众路线教育实践活动、“三严三实”专题教育和“两学一做”学习教育，严格执行中央八项规定，严厉整治慵懒怠政、为官不为等问题，扎实推进党风廉政建设和反腐败斗争，政府执行力、公信力明显增强，风清气正、干事创业的良好氛围更加浓厚。

与此同时，我们深入推进军民融合、军地协调发展，海军某护卫舰被命名为“许昌舰”，建立了首个杨根思拥军社区，第5次荣获“全国双拥模范城”。民族、宗教、外事、侨务、对台工作全面加强，妇女儿童、残疾人、慈善、红十字工作健康发展，统计、史志、档案、气象、地震、人民防空、对口援疆等工作取得新成绩。

各位代表！

经过五年的艰苦奋斗，我市经济社会持续健康发展，综合实力和竞争力大幅提升，城乡面貌显著改变，人民群众得到更多实惠，许昌发展站在了新的更高起点上，为率先全面建成小康社会奠定了坚实基础。这些成就的取得，是省委、省政府和市委正确领导的结果，是市人大、市政协监督支持的结果，是全市广大干部群众担当有为、攻坚克难的结果。在此，我代表市人民政府，向全市广大干部群众、驻许部队官兵、武警指战员、公安民警致以崇高敬意！向所有关心支持许昌发展的社会各界人士表示衷心感谢！

成绩来之不易，经验弥足珍贵，需要倍加珍惜、发扬光大。一是必须旗帜鲜明讲政治。只有坚持党的坚强领导，坚持正确的政治方向，把讲政治贯穿政府工作始终，牢固树立“四个意识”，坚决同以习近平同志为核心的党中央保持高度一致，不含糊、不动摇，营造“山清水秀”的政治生态，才能为发展提供坚强的政治保证。二是必须坚定不移抓发展。只有坚持发展第一要务，突出产业发展这一重点，不懈怠、不折腾，一张蓝图绘到底，一任接着一任干，才能切实维护好、巩固好许昌的良好发展态势。三是必须应时合势抢机遇。只有因势而动、顺势而为，准确把握发展大势、深刻理解大政方针，抢抓机遇，奋发有为，开拓进取，才能抢占先机、赢得主动。四是必须改革创新破难题。只有把改革、开放、创新作为破解发展难题的关键举措，以改革推动发展，以开放带动发展，以创新驱动发展，才能突破瓶颈制约、摆脱路径依赖、拓展发展空间、释放发展活力。五是必须全心全意惠民生。只有坚持以人民为中心，做到一切为了人民、一切依靠人民，努力办好关系群众切身利益的事情，才能赢得人民最广泛的理解、拥护和支持，形成干事创业的强大合力。

在肯定成绩的同时，我们也清醒看到，发展中还存在诸多问题和挑战，主要是：转型升级任务艰巨，传统动能持续减弱，新兴动能尚未形成规模，高新技术产业占比偏低，发展质量效益有待提高；城镇化水平不高，中心城市辐射带动能力不强，城乡区域发展不平衡；教育、医疗、文化、养老等公共服务水平较低，优质资源供给不足；资源环境约束趋紧，生态环境保护任务繁重；各类矛盾风险交织叠加，维护社会稳定压力加大；政府职能转变仍有差距，一些政府工作人员全局意识、责任意识、为民意识、法治意识树得不牢，不作为、慢作为、乱作为等问题仍然存在。民之所望为施政所向，我们将牢记责任使命，坚持问题导向，切实解决突出问题，决不辜负人民厚望。

二、今后五年总体思路和2017年重点工作

今后五年，是许昌转型发展的关键期、改革开放的机遇期，更是率先全面建成小康社会的决战决胜期。做好政府工作，要全面贯彻党的十八大和十八届三中、四中、五中、六中全会精神，深入贯彻习近平总书记系列重要讲话精神和治国理政新理念新思想新战略，统筹推进“五位一体”总体布局和协调推进“四个全面”战略布局，认真贯彻落实市委决策部署，以稳中求进为总基调，以打好“四张牌”为总要求，以供给侧结构性改革为主线，以提高发展质量和效益为中心，以建设“四个强市”为重点，着力推进郑许融合发展，着力推动转型发展，着力加快推进新型城镇化，着力深化改革开放，着力防风险惠民生，促进经济平稳健康发展和社会和谐稳定，努力建设实力许昌、活力许昌、魅力许昌，确保率先全面建成小康社会。

当前，外部环境依然复杂严峻，世界经济增长乏力，全球贸易持续低迷，国内经济“三期叠加”，下行压力依然较大，许昌自身发展同样面临不少矛盾和问题，风险和挑战依然存在。困难不容忽视，信心不可动摇。世界新一轮科技革命和产业变革蓄势待发，我国经济长期向好的基本面没有变，国家推进“一带一路”战略和供给侧结构性改革，我省实施“三区一群”等国家战略，许昌成为郑州大都市区次中心城市，这些都为我市加快发展提供了难得的历史机遇。特别是市七次党代会为许昌未来五年发展描绘了宏伟蓝图，吹响了建设“三力许昌”、“四个强市”、决胜全面小康的集结号。我们坚信，有省委、省政府和市委的坚强领导，全市上下万众一心、奋力拼搏，我市发展一定能够创造新的辉煌。

今后五年的奋斗目标是：在提高发展平衡性、包容性、可持续性的基础上，力争主要经济指标增速高于全省全国平均水平、主要人均指标高于全省全国平均水平。到2021年，全市生产总值完成3500亿元左右，年均增长8%以上；一般公共预算收入突破200亿元，年均增长9%以上；规模以上工业增加值突破2000亿元，年均增长8%以上；居民收入与经济发展同步增长，城乡居民收入进入全省第一方阵，公共服务体系更加健全，人民群众生活质量、健康水平、幸福指数明显提高。经过五年奋斗，全市经济实力和综合竞争优势大幅提升，“一极两区四基地”建设迈出坚实步伐，成为中原城市群发展的重要战略支撑，成为让中原更加出彩的“领头雁”之一。

根据市“十三五”规划和市委经济工作会议确定的发展目标，今年全市经济社会发展的主要预期目标是：生产总值增长8%以上，一般公共预算收入增长8%以上，规模以上工业增加值增长8.5%，服务业增加值增长9.5%，固定资产投资增长12%，社会消费品零售总额增长11%，实际到位省外资金增长7%，进出口总值、实际利用外商投资保持稳定，城乡居民收入与经济增长基本同步，居民消费价格涨幅控制在3%左右；节能减排完成省下达任务。

今年将召开党的“十九大”，是党和国家事业发展中具有重大意义的一年，要重点抓好以下工作：

（一）加快郑许融合发展，开创许昌发展新格局。建设郑州大都市区是国家的大战略、全省的大举措，推进郑许融合发展是许昌发展的大机遇，也是广大群众的大福祉。要把郑许融合作为牵动全局的一号工程和主战略，依托郑州、对接空港、发挥优势、错位发展，在加快融合发展、建设大都市区次中心城市进程中走在前列。

加强规划引领。高标准编制郑许融合发展战略规划、空间布局规划、许港产业带总体规划，做好许东生态城、国家级高新区、高铁北站组团等关键节点规划，统筹生态布局、道路交通等专项规划，着力完善规划体系。

加强功能对接。推动中心城市北进拓展和组团式发展，主动承担大都市区部分金融、科技、文化、教育、旅游、养生、宜居等核心功能，放大综合优势，突出发展特色，实现与郑州互动发展。

加强交通对接。坚持交通先行，协同推进高速铁路、货运铁路、城市快轨、高速公路、快速通道等交通设施建设，加快构建布局合理、功能完善、高效便捷的郑许间立体交通体系，建成许港快速物流通道，年内开工建设机场至许昌快速轨道交通，着力打造与郑州中心枢纽功能互补、紧密衔接、辐射豫南的大都市区门户枢纽。

加强产业对接。以中心城区和长葛市域为重点，强力推动许港产业带建设，重点发展先进制造业、临空产业和高端服务业，致力打造具有国际竞争力的优势产业集群。加快中德（许昌）产业园等项目建设，布局建设一批高端制造业项目，实现优势互补、协同发展。

加强生态对接。依托全市90万亩花木林海，巩固扩大生态环境优势，打造魅力彰显的郑州“南花园”。加快建设国家储备林基地，支持鄢陵打造全域花海，加快京广高铁等生态景观廊道建设，促进生态、健康、养老、旅游产业融合发展，实现生态效益与经济效益、社会效益有机统一。

加强平台对接。深化交流合作，充分利用“三区一群”等国家战略平台，加快综合保税区、保税物流中心和功能性口岸规划建设，推动信息、研发、金融、结算等公共服务平台与郑州对接，实现互联共享，努力构建相互衔接、高效便利的开放发展支撑体系。

（二）促进经济平稳运行，实现经济增长质量新提升。突出稳定工业增长，积极扩大有效投资，促进消费升级，稳定贸易出口，巩固扩大我市良好发展态势。

服务实体经济发展。加强重点行业企业监测预警，深化服务企业工作，一企一策解决企业困难。强化企业资金保障，落实与省级金融机构战略合作协议，力争新增贷款175亿元；通过企业上市、发行债券、投资基金等，实现直接融资100亿元以上；积极开展“银政通”业务，解决企业融资难题。认真落实企业上市培育行动计划，加强政策扶持和全程服务，新增境外上市企业1家、新三板挂牌企业6家以上，新培育壮大一批大公司大集团。

积极扩大有效投资。推动实施“18925”投资促进计划，突出抓好180个重点项目建设，完成年度投资900亿元以上，带动全市固定资产投资2500亿元以上。坚持完善市级领导分包重点项目等推进机制，落实联审联批、模拟审批、并联审批等制度，保障要素供给，加快建设速度。加强重大项目谋划储备，不断增强发展后劲，培育新的经济增长点。

促进消费扩大升级。落实国家、省扩大和促进消费带动转型升级实施方案，实施城镇商品销售畅通等十大行动，进一步放宽服务业准入，加快发展全域旅游、健康护理、体育健身、绿色消费等新型消费业态。加强农村消费基础设施建设，增加高质量、高水平产品有效供给。创建“放心消费城市”，完善消费政策，优化消费环境，加强消费者权益保护，让群众少烦心、多舒心。

扩大外贸出口规模。落实国务院促进外贸回稳向好政策措施，发挥区位和产业优势，优化出口产品结构，建设外贸强市。推动发制品等产业提质转型，扩大电力电气设备、汽车零部件、绿色农产品等出口份额，提高机电产品出口比重，促进出口稳定增长。加快外贸公共服务平台和跨境电商口岸建设，鼓励支持企业开拓国际市场。

（三）打好转型发展攻坚战，构建现代产业新体系。以大数据为引领，以信息化为支撑，以智能制造为抓手，加快调整优化产业结构，突出新兴产业和新业态培育、传统产业转型升级、产业深度融合创新，推动产业向高端化、绿色化、智能化、融合化迈进。

着力建设先进制造业强市。坚持优化存量与扩大增量并举，培育新动能与改造提升传统动能并重，实施中国制造2025许昌行动纲要和“许昌制造7475”工程。开展新一轮大规模技术改造，壮大电力装备等特色优势产业，促进产业向高端化发展。重点培育高端装备制造、新能源汽车、生物医药、新能源等战略性新兴产业，战略性新兴产业占工业的比重超过40%。深入推进制造业与互联网融合发展，加快实施“设备换芯”、“生产换线”和“机器换人”，推广应用智能车间、智能工厂建设模式。鼓励大企业集团加大创新投入、改善经营管理、积极并购重组，完善中小企业公共服务体系，引导中小企业向“专、精、特、优”发展。突出抓好中原硅材料产业园、许昌生物医药产业园等110个工业转型升级重点项目，完成年度投资396亿元。加快产城融合试点市建设，推动产业集聚区提质转型，创建国家和省级高新区。加快国家高新技术产业检验检测基地建设。积极创建全国质量强市示范城市，加强全面质量管理，健全质量竞争机制，创造更多“中国名牌”。

着力建设现代服务业强市。落实“1242”服务业发展计划，积极发展新业态、新模式，打造经济增长新引擎。大力发展康养产业，以生态资源为依托，发挥中医药产业特色优势，促进医养结合，支持现有医疗和养老机构开设新业务，引进国内外优质医疗养老资源，规划建设许昌国家中医药综合改革试验区，重点推动鄢陵县、禹州市、市城乡一体化示范区“一区三园”健康养生养老基地建设。大力发展现代物流业，建设许昌综合物流园区，推动多式联运、冷链物流、城乡邮政、快递运送融合发展，初步建成四级物流配送网络。以冷链物流为龙头，完善交易平台，加快建设集疏中心、生产加工、仓储设施和冷链装备制造等冷链项目，打造中原地区最大的“冷谷”。大力发展现代金融业，积极引进金融机构，支持发展地方中小金融机构和县（市）分支金融机构，支持金融组织、产品和服务创新，规划建设市商务中心区金融中心，增强金融综合服务能力。突出发展信息服务业，加快市、县两级电子商务产业园建设，培育壮大发制品、蜂产品等特色电商产业，全市电商年交易额突破500亿元。大力发展文化旅游业，加快魏都区、鄢陵县国家全域旅游示范区建设，推进神垕国家级特色小镇等项目，积极创建国家级旅游度假区，力争新增1—2家4A级景区。加快服务业“两区”提速增效，实现产业集聚、特色培育和功能提升，促进总部经济、文化创意等业态集聚，加快现代服务业核心区开发和商业综合体项目建设，打造东城区商圈。

着力建设现代农业强市。以增加农民收入、强化有效供给为主要目标，聚焦“四优四化”，实施“五大工程”，推进农业供给侧结构性改革，培育农业农村发展新动能。落实“藏粮于地、藏粮于技”战略，保护和提高粮食综合生产能力，完成435万亩永久基本农田划定任务，新建和改造提升高标准粮田20万亩，新增高效节水灌溉面积17万亩，粮食产能稳定在280万吨以上。优化农业生产结构和产品结构，推广种植优质专用小麦40万亩，压减玉米种植20万亩，适度扩大花、菜、药、烟、菌、薯等优势特色产业规模；促进生态循环畜牧业发展，积极创建国家级绿色畜牧业示范县和标准化示范场；推行绿色生产方式，增加绿色优质农产品供给。支持发展多种形式的适度规模经营，大力培育新型职业农民和农业企业家，发展专业大户、家庭农场、农民合作社等新型经营主体1000家以上。大力发展都市生态农业和休闲农业，积极培育都市生态农业示范园区和特色示范基地。发展农产品精深加工业，延长农业产业链和价值链。鼓励和支持发展外向型农业，全面提高现代农业外向度。促进农业与旅游、教育、文化、健康等产业深度融合，发展新业态新模式，广辟农民增收致富门路。探索“资源变资产、资金变股金、农民变股东”模式，盘活资产，增加农民群众财产性收入。

着力建设网络经济强市。深入实施“互联网+”行动计划，促进数字经济加快成长，让企业和群众普遍受益。大力实施光纤网络改造提速工程，落实国家降低网络资费政策，全面提高宽带用户平均接入速率。以国家大数据综合试验区建设为契机，推进大数据产业园、政务云和公有云计算中心建设，加快数据资源和信息系统互联互通，促进“政产学研用”联动、大中小企业协调发展，形成大数据共享采集、开发使用体系。将物联网技术逐步融入物流运输、电网调度、智能装备等重点领域。推动跨境电商与农村电商有机结合，加快鄢陵县国家级电子商务进农村示范县建设。完善网络安全保障体系，提高信息安全保障水平。

（四）实施创新驱动战略，培育经济发展新动能。科学编制《许昌市创新驱动发展战略规划》，着力提高全要素生产率，让创新成为引领许昌发展的第一动力。

壮大创新主体。突出抓好企业创新，培育一批创新引领型企业，壮大一批高新技术企业，发展一批科技型中小企业，增强全社会持久创业创新动力。新培育高新技术企业20家以上、创新型试点企业15家、知识产权优势企业2—3家。深入开展产学研合作，积极争取科研院所和高等院校对企业技术创新的源头支持，努力在引进大院名校上迈出实质性步伐。

汇聚创新人才。积极实施“许昌英才计划”，优化人才发展环境，突出“高精尖缺”导向，培育引进一批创新引领型人才团队。新组建一批院士工作站和博士后科研流动工作站。实施更加有效的人才引进政策，畅通高层次高技能人才引进绿色通道，建立领军型人才柔性引进机制，改进人才评价体系，吸引更多优秀人才到许昌创新创业。

构建创新平台。建设一批创新引领型平台，争取国家级高新区获批。以创建国家创新型城市为抓手，新建省级创新研发平台3—5个，新建市级以上创新研发平台30个，支持行业龙头企业组建产业技术创新战略联盟。做大做强众创空间和科技企业孵化器，激发全社会创业创新潜能。

完善体制机制。积极利用国内外高端创新资源，深化产教融合和校地校企合作，形成全方位开放创新格局。加强知识产权创造、保护、运用和管理，深化科普和创新文化建设。落实创新支持政策，完善科技创新投入和奖励制度体系，让创新创造蔚然成风。

（五）扎实推进新型城镇化，丰富城市发展新内涵。坚持以人为核心，以建设中原宜居城市为目标，着力提高城镇化发展质量，推动城乡区域协调发展。

加强中心城区建设。以新一轮城市总体规划为引领，优化发展举措，破解发展难题，厚植发展优势。推进实施“城市提质1421工程”，重点抓好220个建设项目，完成年度投资353亿元。加快推进曹魏古城、城市中轴开发和市文化艺术中心、市体育中心等项目，不断完善城市功能。提高城市文化品位，突出曹魏文化特色，彰显城市文化内涵。加快市城乡一体化示范区建设，促进产城融合发展。统筹新城建设与旧城改造，提升中心城区西部区域发展。以创建“中国人居环境奖”为统领，深入开展“五城联创”，巩固提升创建成果。整治城区易涝区段，有效治理交通拥堵等“城市病”。加强中心城区河湖水系管护及周边环境整治，完善水系配套设施，让清水长流、碧波永驻，进一步提高许昌的宜居性和美誉度。

大力实施百城建设提质工程。围绕增强综合承载能力、推进产城融合发展、改善人居环境、提升精细化管理水平等，提高规划编制水平，强化顶层设计，科学确定城市定位，精心塑造城市特色，充分彰显城市魅力，逐步实现从拉大框架向提升质量转变。全面落实许昌城市发展“1+5”PPP投融资协议，加快推进580个总投资2070亿元的百城建设提质工程项目，谋划实施一批基础设施和公共服务项目。加快县级城市内涵式发展，加大“三城联创”力度，打造和谐宜居、富有活力、各具特色的现代化县城。

推进农业转移人口市民化。深化户籍制度改革，完善配套政策体系，加快构建以居住证制度为载体的基本公共服务供给机制，让落户城市的人“进得来、留得住、过得好”。坚持产业为基，以产业带动就业，为农民进城创造条件。进一步扩大住房保障范围，完善农业转移人口住房保障体系。扩大教育资源供给，保障随迁子女平等享有教育权利。深化农村产权制度改革，完成农村土地确权登记颁证任务。支持禹州、长葛国家新型城镇化综合试点市建设。

提高房地产业发展质量。坚持“房子是用来住的，不是用来炒的”定位，加强舆论引导，稳定市场预期。实施动态调控机制，加强房地产市场形势研判和监管，科学把握住宅用地供应节奏。优化房地产增量，实现多样化商品房供给，引导和规范发展养老地产、旅游地产等新型地产。推进建筑业改革发展，提高设计水平和工程质量。建立建筑企业信用评价体系，引导龙头骨干企业组建联合体参与市场竞争。实施棚户区改造三年攻坚计划，今年启动改造项目44个，新开工3.16万套，完成年度投资90亿元，真正让群众成为棚户区改造的受益者而不是受害者。

完善现代交通体系。加快许昌境内郑合、郑万高铁及4个高铁站建设，抓好国道107线许昌境改建、国道311线许昌境改建、新元大道东延、忠武路及地下综合管廊等重大交通设施建设，加强运输场站和交通物流园区建设，完善公路运输和服务网络。倡导绿色出行，优先发展公共交通，积极争创国家公交都市。

加快中心镇和乡村建设。实施重点镇建设示范工程，支持神垕、大周、陈化店、五女店等重点特色小镇加快发展。新建和改造提升农村公路620公里，新增农村自来水受益人口34.2万人，完成中心村和贫困村电网改造目标任务。统筹解决新型农村社区建设遗留问题。加强农村环境综合治理，大力实施乡村清洁工程，打造美丽宜居乡村。加强古村落保护，延续文脉，留住“乡愁”。

（六）全面深化改革开放，激发经济社会发展新活力。坚持改革推动、开放带动，鼓励支持民营经济发展，增强内生动力，增添发展活力。

深化重点领域改革。深入推进供给侧结构性改革，认真落实“三去一降一补”重点任务，坚决淘汰不达标落后产能，退出煤炭产能306万吨；建立健全降成本工作协调机制，全面落实减税、降费、降低要素成本等政策措施。坚持市场化改革方向，扎实推进“放管服”改革，再取消或下放一批审批事项，加快推进“互联网+政务服务”，着力打通为民服务“最后一公里”。完善“双随机、一公开”监管机制，实现行政许可、行政处罚信息“双公示”全覆盖。持续打好国企改革攻坚战，妥善化解历史遗留问题。加快商事制度改革，探索试行“多证合一”，开展减证便民专项行动。深化金融体制改革，探索发展各类产业基金，筹建森源集团金融租赁公司。深化财税体制改革，优化支出结构，完善财政资金投入机制。抓好投融资体制改革，加快政府投融资平台市场化转型，建立多元融资制度，积极推动PPP项目，全面提升市、县两级投融资平台融资能力，争创省级投融资试点城市。全面推进公立医院综合改革，开展分级诊疗试点，推广家庭医生签约服务。全面完成城市管理体制改革。

不断扩大开放招商。抓住“一带一路”建设机遇，巩固扩大与欧盟、东盟成员国合作成果，深化对德合作。坚持引资引智引技并重，实现“招商”向“选商”转变，瞄准国内外500强、央企和行业龙头企业，创新招商方式，办好专题招商，注重项目落地，提高招商实效。改善投资环境，维护市场公平，保护投资者合法权益。鼓励富余优势产能“走出去”，参与国际竞争与合作。

大力发展民营经济。深入落实支持非公有制经济发展的政策措施，对民营企业坚持权利平等、机会平等、规则平等。加大支持保障力度，建立完善政策激励、企业风险化解机制。用足用活金融工具，清理规范涉企收费，发挥担保机构作用，帮助民营企业解决融资难题。扎实推进“企业家队伍培养十百千行动计划”，壮大现代企业家群体。进一步优化政务环境，着力营造“亲”“清”政商关系。

（七）加强生态文明建设，厚植绿色发展新优势。绿水青山就是金山银山。以生态文明建设为统领，以国家生态文明先行示范区建设为载体，统筹生产生活生态，科学施策，标本兼治，铁腕治理，让许昌天更蓝、水更清、环境更优美。

实施环境治理攻坚。保持大气污染防治高压态势，强化扬尘和散煤治理、工业污染源监管、禁烧禁放等重点措施，有效应对重污染天气，确保PM10、PM2.5浓度稳定下降，空气优良天数207天以上，全面完成省定任务。加强水污染治理，全面实行“河长制”，坚持一河一策、水陆统筹，系统推进水污染防治、水生态保护和水资源管理，强化饮用水水源地保护，提升水环境质量，彻底消除县（市）城区黑臭水体。制定土壤污染防治行动计划，启动土壤污染状况详查，有效防控污染土壤环境风险。让群众呼吸上清新的空气、喝上干净的水、吃上安全的食品。

加强林业生态建设。实施“绿满许昌”行动计划，推动森林、湿地、流域、农田、城市“五大生态”建设，加快山区、矿区、河道生态修复和平原绿化进程，加强城郊森林生态体系建设，积极构建生态廊道网络。全面提升村镇绿化美化水平，打造20个都市生态农业（林业）园，形成半小时城郊休闲观光圈。完成林业生态建设任务12.56万亩，打造结构稳定、功能完善的绿色屏障。

推进低碳循环发展。积极发展绿色产业，倡导绿色生活方式，在全省率先实行绿色建筑全覆盖。支持新能源汽车产业发展，加快电动汽车充电设施建设。实施全民节能行动计划，提高工业、建筑、交通运输、公共机构等领域能效水平。大力发展循环经济，加快循环经济示范县（市）、循环经济示范园区和静脉产业园建设，构建循环型产业体系，实现环境改善与经济发展双赢。

（八）切实保障和改善民生，践行共享发展新理念。从解决群众最关心最直接最现实的利益问题入手，竭力抓好各项民生工程和民生实事，织密织牢民生保障网。

坚决打赢脱贫攻坚战。把脱贫攻坚作为重要政治任务和第一民生工程，牢记使命担当，树立为民情怀，把握“精准”核心，下足“绣花”功夫。认真落实“1+N”政策体系，严格对照“两不愁三保障”标准，坚持“六步工作法”，做到精准识别、精准帮扶、精准退出，完成44个村、2.17万贫困群众脱贫任务。按照“六个一”的要求，全面查摆整改存在问题，着力抓好档案资料规范化建设，确保实现“三个零差错”、“三个显著提高”，一次通过复检、走在全省前列。把产业扶贫作为脱贫攻坚的治本之策，因地制宜发展种植业、养殖业、农村电商、光伏发电等产业，确保脱贫不返贫，在扶贫路上决不丢下一户、落下一人。

大力促进就业创业。认真落实高校毕业生就业创业促进计划，加大对农村富余劳动力、就业困难人员、贫困家庭劳动力、去产能企业分流职工等重点群体就业帮扶力度，做好军队转业干部、退役士兵接收安置工作，促进“零就业家庭”成员实现就业。促进以创业带动就业，统筹高校毕业生、返乡农民工、专业技术人员创新创业，完善创业担保贷款制度，打造大众创业高地。继续推进全民技能振兴工程，开展各类职业技能培训10万人次以上，全市新增城镇就业、农村劳动力转移就业各6万人，让广大群众既安居又乐业。

发展更高质量的教育。完善城乡义务教育学生“两免一补”政策，全面实施基础教育提升三年攻坚计划，扎实推进“全面改薄”和职教攻坚，加快建设城镇义务教育学校扩容工程，今年新建改扩建幼儿园50所以上、中小学100所以上。加快职教园区、许昌二高二期、许昌高中北校区改建等重点项目建设。以优质均衡为导向，加强师资队伍建设，提高教育质量，培育和引进更多名师、名校。落实教育经费法定增长要求，完善教育投入机制，逐步提高教师待遇。深化集团化办学，快速放大优质教育资源。加大政策扶持力度，引进优秀管理团队，加强对外合作，鼓励社会力量办学，支持学校多样化发展，满足群众特色教育需求，让许昌的孩子在家门口就能享受优质教育，让更多外地人慕名来许昌求学。

加强公共文体服务。构建现代公共文化服务体系，支持市级媒体融合发展，推进市融媒体产业发展基地、基层综合性文化服务中心建设，积极创建全国公共文化服务示范区。启动灵井“许昌人”遗址公园规划建设。加强文艺精品创作生产，继续实施文化惠民工程，丰富群众文化生活。广泛开展全民健身，投资1.2亿元，新增乒乓球、篮球、羽毛球等公共体育设施和儿童游乐设施398处（套），着力打造“十五分钟”健身圈。

加快“健康许昌”建设。实施《“健康许昌2030”行动规划》，为人民群众提供全方位、全生命周期的卫生与健康服务。加快市中心医院新院区、市妇幼保健院整体迁建等11个卫生项目建设。加强与省内外优质医疗机构合作，柔性引进和大力培育高素质医疗人才，大幅提升医疗服务能力。建立市、县、乡三级救治网络体系，打造中心城区半小时、市域范围2小时急救圈。加强中医药文化宣传，发展中医药健康服务，促进中医药健康服务能力大幅提升、产业规模不断扩大。加强基层医疗卫生机构建设，新建和规范提升社区卫生服务中心7个，实现村级标准化卫生室全覆盖。实施改善医疗服务行动计划，提高人民群众就医满意度。注重预防为主和健康促进，加强重大疾病、传染病和慢性病防控。支持社会资本办医，满足人民群众多层次、多样化的医疗卫生服务需求。加强乡村医疗用药监管和技术指导，提高乡村医生待遇水平。改革完善计划生育服务管理，落实“全面两孩”政策，促进人口长期均衡发展、家庭和谐幸福。

增强社会保障能力。实施全民参保登记计划，推进社会保险“五险合一”，加快城乡居民基本医疗保险制度整合，继续提高退休人员基本养老金标准，扩大各项社会保险覆盖面。推动农村低保与扶贫政策有效衔接，落实困难残疾人生活补贴和重度残疾人护理补贴制度，城市低保标准提高到每人每月不低于450元，农村低保标准提高到每人每年不低于3300元。加强公租房管理，降低准入门槛，简化申报程序，让住房困难群众及早入住。完善社会救助和社会福利体系，关心帮助军烈属和孤寡老人，让困难群众求助有门、受助及时。

确保社会大局和谐稳定。严格落实安全生产责任，深入开展安全生产大检查、“打非治违”、隐患排查治理等活动，抓好重点领域专项整治，坚决防范和遏制重特大安全事故发生。健全食品药品安全监管体制机制，继续抓好国家食品安全示范城市创建工作。加强政府债务管控，完善金融监管体制，依法打击各类金融违法行为，制定防范化解金融风险预案，坚决守住不发生系统性金融风险底线。继续治理非法集资、问题楼盘、信访积案三类突出问题，落实信访工作责任制，建立社会矛盾多元化解机制，及时排查调处各类纠纷。加强防灾减灾救灾，建立健全突发事件预警信息发布体系。推进社会诚信体系建设，努力营造守信光荣、失信可耻的良好社会氛围。深化平安许昌建设，健全立体化、信息化社会治安防控体系，严厉打击各类违法犯罪活动，大力整治社会治安突出问题，全方位提高人民群众安全感。

加强国防动员、国防教育和国防后备力量建设，深入开展双拥共建，推进军民深度融合发展，持续创建“全国双拥模范城”。支持工、青、妇等人民团体更好发挥桥梁纽带作用，促进民族团结进步、宗教关系和谐，全面加强外事、侨务、港澳、对台工作。扎实做好统计、史志、档案、社科研究、气象、地震、对口援疆等工作。

（九）全面加强政府自身建设，提高科学发展新能力。坚持党的领导、人民当家作主、依法治国有机统一，殚精竭虑，勤勉工作，永不懈怠，努力建设法治政府、高效政府、廉洁政府和人民满意政府。

坚持依法行政。强化法治意识，全面贯彻法治政府建设实施纲要，完善推进机制，把依法行政、依法办事的原则和要求贯穿于政府立法、决策、执行的全过程。切实改进政府立法，严格行政执法，加强执法监督，依法全面履行政府职能。推动各级政府及其工作人员深入贯彻全面依法治国要求，严格遵守宪法，尊崇法治、敬畏法律、依法行政，自觉运用法治思维和法治方式深化改革、推动发展、化解矛盾、维护稳定，把政府活动全面纳入法治轨道。

坚持执政为民。强化宗旨意识，始终做到以人民为中心，自觉把人民群众的冷暖放在心上，办好顺民意、解民忧、惠民生的实事。加强政务服务体系建设，为群众和市场主体提供优质便捷高效服务。依法接受人大法律监督和工作监督，自觉接受政协民主监督，认真办理人大代表议案、建议和政协提案，认真听取民主党派、工商联、各人民团体和各界人士的意见建议，自觉接受人民监督。

坚持务实重干。强化发展意识，求规律之真，务发展之实，不做表面文章，不求数字好看，不动摇、不折腾，脚踏实地抓落实，撸起袖子加油干。健全激励机制和容错纠错机制，给干事者鼓劲，为担当者担当。完善常态化督查问责机制，严厉整肃庸政懒政怠政行为。团结带领广大干部主动作为，发扬拼劲、狠劲和韧劲，以实干推动发展，以实干赢得未来。

坚持清廉为政。强化廉洁意识，认真落实全面从严治党要求，把党风廉政建设和反腐败工作不断引向深入。增强“四个意识”，强化政治担当，落实“一岗双责”，切实承担起党风廉政建设主体责任。推进“两学一做”学习教育常态化制度化。严明政治纪律和政治规矩，严格执行《准则》和《条例》，强化正风肃纪，坚决惩治侵害群众利益的不正之风和腐败问题。严格落实党中央八项规定和省委、省政府20条意见，一以贯之纠正“四风”，厉行节约，反对浪费，降低行政成本。加强行政监察和审计监督，始终保持惩治腐败高压态势，坚持“无禁区、全覆盖、零容忍”，以反腐败的实际成效保障发展、取信于民。

各位代表！

展望未来，任重而道远，我们深感责任重大、使命光荣。让我们更加紧密地团结在以习近平同志为核心的党中央周围，在省委、省政府和市委的正确领导下，紧紧依靠全市人民，振奋精神，凝神聚力，真抓实干，克难攻坚，为加快建设实力许昌、活力许昌、魅力许昌，率先全面建成小康社会而努力奋斗，以优异成绩迎接党的十九大胜利召开！
